# Draft Genome Sequences of Marine Isolates of *Thalassomonas viridans* and *Thalassomonas actiniarum*

**DOI:** 10.1128/genomeA.00297-15

**Published:** 2015-04-16

**Authors:** Israel Olonade, Leonardo Joaquim van Zyl, Marla Trindade

**Affiliations:** Institute for Microbial Biotechnology and Metagenomics (IMBM), University of the Western Cape, Cape Town, South Africa

## Abstract

*Thalassomonas viridans* and *Thalassomonas actiniarum* are aerobic Gram-negative bacilli which belong to a genus that has not received much attention, even though, as demonstrated here by the sequencing of their genomes, they are quite different from their closest relatives in current databases. Their genomes are relatively large at 7.7 and 7.4 Mb, respectively. This brief report describes the first draft genomes for any *Thalassomonas* species.

## GENOME ANNOUNCEMENT

Here, we present the genome sequences of *Thalassomonas viridans* (XOM25^T^) and *Thalassomonas actiniarum* (A5K-106^T^)*.* Currently, there are no genome sequences available for any members of this genus, of which the genomic content appears to be novel with average nucleotide identity of 81% and 80% for *T. viridans* and *T. actiniarum* (85% to each other), respectively, when compared to *Colwellia psychrerythraea*, identified as their closest relative for which a genome sequence is available (Kostas lab tools; see http://tinyurl.com/pa2w48l). As the strains sequenced here are also the type strains for each species, the genome sequences represent an important foundation for further research on these poorly studied bacteria. The closely related *Thalassotalea loyana* (formerly *Thalassomonas loyana*) was shown to be the causative agent of plague-like disease of corals on the Eilat coral reef (1). *T. viridans* was isolated from cultivated oysters off the coast of Spain (2), while *T. actiniarum* was isolated from a sea anemone off the coast of Japan (3); although not identified as pathogens of these hosts, *T. viridans* and *T. actiniarum* may play a role in their health as part of their natural microflora.

DNA was prepared using standard proteinase K/SDS treatment, followed by purification using phenol/chloroform extraction. The DNA was further purified using the Qiagen Qiaex II gel extraction kit prior to sequencing. Sequencing was performed on an Illumina MiSeq, using the Nextera XT kit for library construction and the MiSeq reagent kit version 3 (600 cycle). The raw reads were trimmed and demultiplexed, and genome assembly was performed using CLC Genomics Workbench version 6.5. Sequencing produced 554,000 paired-end reads (forward and reverse) for *T. viridans*, with an average read length of 280 bp. Contigs ≤500 bp were removed from the final assembly, giving a total of 286 contigs with an *N*_50_ value of 62,266 bp and the largest contig being 225,113 bp. The draft genome is 7,713,293 bp (48.9% G+C content) with an estimated coverage of 25×. For *T. actiniarum*, 1,031,082 reads were generated at an average read length of 239 bp. The *N*_50_ value was 71,374 bp, with the longest contig being 426,993 bp. The draft genome assembled to 132 contigs ≤500 bp, totaling 7,420,947 bp (47.3% G+C content) with an estimated coverage of 36×. A basic annotation was performed using the RAST server (4). *T. viridans* has 6,667 (2,476 hypothetical) coding sequences and 76 tRNAs, while *T. actiniarum* has an estimated 6,481 (2,379 hypothetical) coding sequences and 96 tRNAs. Analysis using the antiSMASH secondary metabolite prediction server (5) showed that both strains contain several, potentially novel, nonribosomal peptide and polyketide biosynthetic clusters (or hybrids thereof), as well as bacteriocins and lantipeptides. At ±7.4 Mb, these genomes are roughly 2 Mb larger than their closest relative and thus could harbor interesting metabolic pathways as well as shed light on their relationship with the hosts from which they were isolated.

### Nucleotide sequence accession numbers. 

These whole-genome shotgun projects have been deposited at GenBank under the accession numbers JYNI00000000 and JYNJ00000000.
